# High Prevalence of Proposed Müllerian Duct Remnant Cysts on the Spermatic Duct in Wild Eurasian Otters (*Lutra lutra*) from Sweden

**DOI:** 10.1371/journal.pone.0084660

**Published:** 2013-12-23

**Authors:** Anna M. Roos, Erik O. Ågren

**Affiliations:** 1 Department of Environmental Research, Swedish Museum of Natural History, Stockholm, Sweden; 2 Department of environmental toxicology, Uppsala University, Uppsala, Sweden; 3 Department of Pathology and wildlife diseases, National Veterinary Institute, Uppsala, Sweden; Temasek Life Sciences Laboratory, Singapore

## Abstract

The spermatic ducts (*vasa deferentia*) of 235 otters (*Lutra lutra*) found dead between 1999 and 2012 in Sweden were examined for presence of paraductular cysts. Single or multiple elongated uni- or bilateral cysts parallel to the spermatic duct were noted in 72% of the examined males. The cysts were adjacent to, but did not communicate with the lumen of the spermatic duct, and were usually located within a few centimeters of the testis and epididymis. The cysts are proposed to be congenital Müllerian duct remnants. Other morphologic abnormalities in the reproductive organs were not noted within this study. Possible causes of the incomplete regression of the embryonic female gonadal duct are exposure to environmental contaminants such as elevated concentrations of estrogen-like compounds (endocrine disrupting chemicals), inbreeding, or a naturally occurring anatomic defect. No obvious geographical pattern was observed for otters with or without cysts. This is the first study and description of cysts on the spermatic duct in otters.

## Introduction

Increasing developmental abnormalities and lesions on the reproductive tract have been observed in a number of species, including humans, for example testicular cancer, cryptorchidism (undescended testis), hypospadias (congenital malformation of the urethra), poor testicular development and function, disorders of sex differentiation and decreased semen quality [1–3]. Furthermore, there is evidence that these male reproductive disorders can be caused by endocrine disrupting chemicals (EDCs) during fetal stage. 

A well-known example is the alligator (*Alligator mississippiensis*) in lake Apopka, Florida, which was heavily exposed to DDT and dicofol. The males from this contaminated lake had significantly lower testosterone levels compared to males in a non-contaminated lake, and they developed poorly organized testes and had abnormally small penises [4,5]. In polar bears (*Ursus maritimus*), there was a significant inverse relationship between organohalogens and testis and baculum size as well as a decreased baculum mineral density correlated to elevated concentrations of some organochlorines [6]. Organochlorines may also affect testosterone levels [7] and the steroid hormone cortisol concentrations [8] in the blood of polar bears. Furthermore, cryptorchidism was found in 16% of the examined black bears (*Ursus americanus*) in Florida [9]. Many Florida panthers (*Felis concolor coryi*) had abnormal sperm, low sperm density or cryptorchidism. There was no significant difference in serum estradiol levels between the sexes indicating that many of the males were de-masculinized/feminized. One possible explanation behind this finding could be exposure to EDCs [10], but also inbreeding has been shown to be part of the reproductive disturbances in the panthers [11].

There are reports that cryptorchidism and other changes on the male reproductive system is increasing in humans [12], most possible correlated to exposure to EDCs [13]. 

Fetal and neonatal periods are specifically sensitive to EDC. The EDC interfere with natural hormones and if exposed during a crucial stage of development they can cause severe and lifelong effects. Some male reproductive endocrine disorders are caused by male hormone insufficiency and/or by an imbalance between male and female hormones during a critical time period. In a study on rats treated neonatally with estrogens it was concluded that the spermatic duct is a main target organ for these substances [14]. 

Since 1972 otters found dead in Sweden have to be reported to the authorities, and carcasses are to be submitted for necropsy and sampling at Swedish Museum of Natural History (SMNH) or National Veterinary Institute (NVI) [15]. Various tissues are sampled for environmental contaminant studies and stored in the Environmental Specimen Bank at SMNH. At diagnostic necropsies, many males were observed to have one or several cysts on the spermatic duct, leading from the testes to the penis. Cysts on the spermatic ducts in humans and in other animal species can have various origins that can be difficult to pinpoint, but are usually presented as being remnants of embryonic female gonadal ducts (Müllerian ducts) that have not undergone full regression [16]. The Müllerian duct system is present in both sexes of the mammalian embryo, but in males it degenerates during sexual differentiation. Anti-Müllerian hormone (AMH) induces the regression of the Müllerian ducts in male fetuses during sex differentiation. The timing of the AMH action on these ducts is critical. In dogs (having similar length of gestation period as otters) the earliest evidence of Müllerian duct regression in male embryos was observed at 36 days gestation, and the regression was completed by day 46 indicating the window of time when the sex differentiation takes place [17]. 

This study describes the prevalence and the detailed anatomy of paraductular cysts, and discusses possible causes for the seemingly high prevalence of these proposed Müllerian duct remnant cysts in Swedish wild male otters.

## Materials and Methods

Male otter carcasses collected between 1999 and 2012 were investigated for the presence of cysts at necropsy at the National veterinary institute in Uppsala or at the Swedish Museum of Natural History in Stockholm. Most otters originated from 2005 - 2012, only 13 were from 1999-2004. A more detail description of the otters is found in Table S1. No otters were killed for the purpose of this study, and no permits were required for the described study, which complied with all relevant regulations. 

The otters were divided into three age groups according to their estimated age: juvenile (up to 5 months old), subadult (approximately 5 -18 months old) and adult (over 18 months old), based on the presence/absence of long bone growth plates, and in some cases also on the size of the testis and baculum [18,19]. If growth plates at the proximal and distal ends of the femur were present, the otter was considered to be a subadult. 

The spermatic ducts (right and left) and testes were evaluated separately. The number, length (mm) and shape of cysts on each duct were recorded. Selected duct samples with cysts were fixed in 10% buffered formalin, trimmed, dehydrated, and placed in paraffin blocks. Approximately five micron sections were cut, mounted on glass slides and stained with hematoxylin and eosin staining method. Randomly selected testes were examined in light microscopy with the same method and stain.

Linear regression analysis was used to test if the prevalence had changed over the years 2005-2012.

## Results and Discussion

Of 235 male otters examined, 154 were classified as adults, 76 as subadults and 5 as juveniles (Table S1). The main causes of death were traffic accidents or drowning in fishing gear. For some otters the cause of death was not possible to determine, usually due to advanced autolysis, however, presence of cysts could still be noted. 

### Anatomy of cysts on spermatic cords

**Figure 1:**
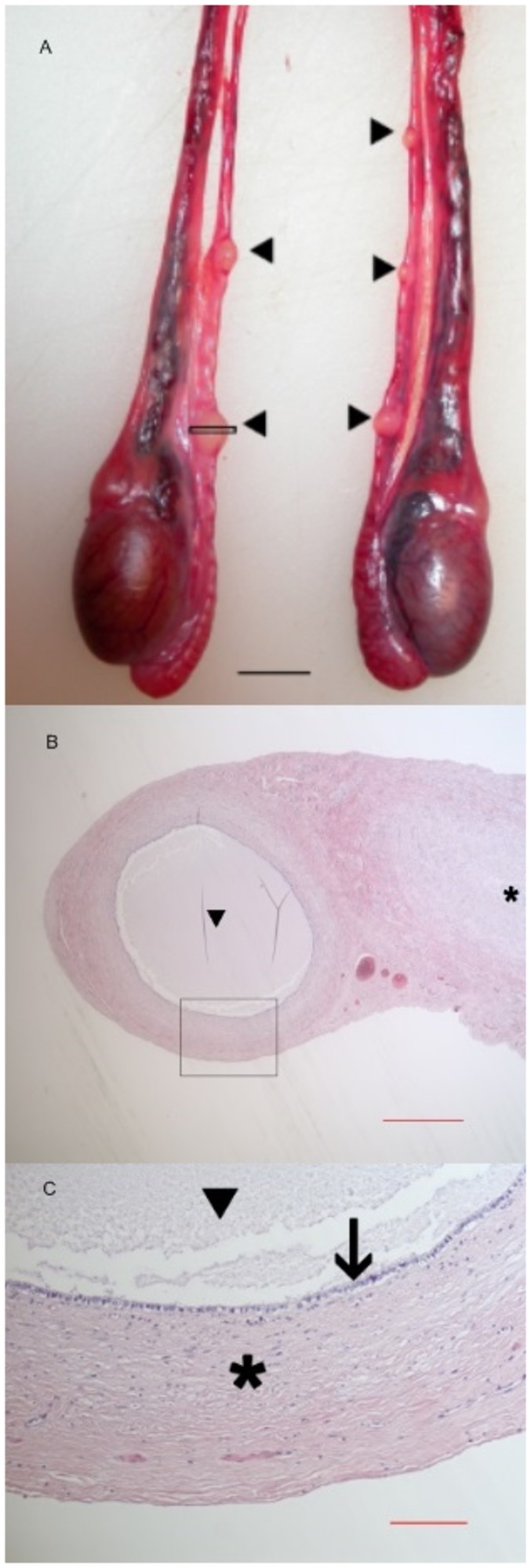
A) Gross anatomical and photomicrographs of Müllerian duct cysts along the spermatic ducts (vasa deferentia) in an otter (*Lutra lutra*). Otter testes, epididymides and funiculi are shown. Arrowheads point to multiple proposed Müllerian duct remnant cysts attached to the spermatic ducts cranial to both the right and left testis. The rectangle indicates were microphotograph in Figure 1b is taken from. Horizontal bar: 1 cm. **B) Microphotograph of a paraductular cyst found in an otter (*Lutra lutra*).** Overview of typical cyst, transversely cut, with fluid filled lumen (arrow head) and no communication to the adjacent vas deferens (asterisk). H&E stain, horizontal bar in the order of 1,5 mm. The rectangle indicates were microphotograph in Figure 1c is taken from. **C) Microphotograph of a paraductular cyst found in an otter (*Lutra lutra*).** Higher magnification of the cyst wall, showing the fibrous capsule (asterisk), internally lined by a single layer of cuboidal epithelial cells (arrow). The cyst contains cell-free granular fluid (arrow head). In some cysts these cells are attenuated and flattened. H&E stain, horizontal bar in the order of 300 µm.

The cysts were located on the spermatic duct, cranial to the epididymus. They were of variable size and measured from 1 mm to 10 mm in length. The cyst shape was usually round to elongated, as larger cysts had a tubular appearance. Elongated cysts and multiple cysts were most often lined up in a row, along the duct, either adjacent to each other or separated by varying distances, parallel to the spermatic duct. This anatomical feature indicated that the cysts rather were remnants of a tubular structure, than an acquired spherical cystic structure expanding from one point source (Figure 1a-c). The cysts had a smooth outer surface, were unilateral or bilateral, and the numbers varied from one to several cysts on one or both spermatic ducts. Serial sections of cysts showed that there was no communication between the cyst and the lumen of the directly adjacent spermatic duct. The cysts had a thin fibrous capsule blending with the surrounding common ligament containing the spermatic duct, and the cysts contained a clear watery fluid. Microscopically the fluid was clear and cell-free. The internal side the capsule was lined with a single layer of mostly attenuated and flattened, and sometimes cuboidal epithelial cells. Occasionally, well-preserved cuboidal cells had cilia-like structures on the luminal surface. The testes were all apart from 7 individuals (4.5 %) located in the expected scrotal area among the adults. Selected samples of testes showed normal architecture and sperm production at histopathology. 

### Frequency of cysts

The prevalence of otters with one or more cysts was 72% (with a 95% confidence interval of: 65-78% assuming binominal distribution). The number of cysts on each spermatic cord varied from one to over ten (Table 1). Unilateral cysts were seen in 49% and bilateral cysts in 51% of otters with cysts. Regarding every individual otter with one or more cysts present (n=167); 37, 28.6, and 34.4% had one, two, or three or more cysts, respectively. Cysts were usually located within 40 mm from to the testis, measured from the cranial border of the plexus pampiniformis. 

**Table 1:** Number of investigated male otter spermatic ducts between 1999 and 2011, presented with number of cysts present, and number of otters divided into the age classes: juvenile, subadult and adult.

**No of cysts**	**Juvenile**	**Subadult**	**Adult**	**Total**
0	4	33	31	68
1	1	20	41	62
2	0	11	35	46
3 or more	0	12	47	59
**Total**	5	76	154	235

**Figure 2:**
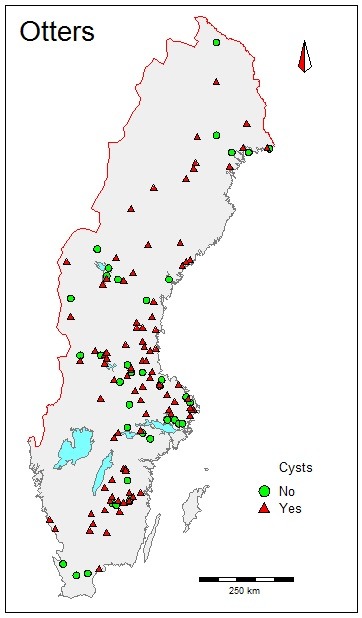
Map of Sweden, showing finding sites of dead otters (*Lutra lutra*) with one or more cysts on the spermatic ducts (red triangles) and otters without any cysts (filled green circles).

Between 2005 and 2012 the yearly frequency of otters with cysts were fairly constant and varied between 63 and 81%. No significant trend was seen (p<0.71) within this short time period. Otters with cysts were found in all studied areas of Sweden and no general geographic pattern can be seen (Figure 2) i.e. they were not more commonly found in more industrialized areas. 

### Etiology

The presence of this type of cyst has to our knowledge not previously been described in otters. We suggest that the cysts are Müllerian duct cysts, which are fetal remnants of the female gonadal ducts that normally regress under hormonal influence, once male differentiation is initiated. Anti-Müllerian hormone (AMH) induces the regression in male fetuses of the Müllerian ducts during sex differentiation and the timing of the AMH action on these ducts is very critical. Further immunohistochemistry studies with CD10 antibodies could possibly differentiate Müllerian ducts from Wolffian duct origin, as in humans [20]. Presently, the CD10 antibody immunohistochemical staining properties have not yet been validated in otter tissues. 

In a laboratory study, pregnant mice were exposed to a synthetic estrogen – diethylstilbestrol (DES). Prominent Müllerian remnants were observed in 97% of the male offspring. These Müllerian remnants were often enlarged and cystic [21]. Therefore, a possible underlying cause to be considered for the cysts in otters are EDCs.

Numerous endocrine disrupting chemicals are found in the environment. Some of them give effect even at low doses in laboratory studies, for example chlordane, Bisphenol A, dioxins, DDT, heptachlor, hexachlorobenzene, nonylphenol, octylphenol, PCBs and polybrominated diphenyl ethers (PBDEs) [22]. Residue levels of many these compounds have yet not been analyzed in otters. In laboratory studies perfluorooctane sulfonic acid (PFOS) and perfluorooctanoic acid (PFOA) have been shown to be potential developmental toxicants and are suspected endocrine disruptors resulting in lower testosterone levels and higher estradiol levels in adult rats [23]. This is interesting considering the increasing and elevated concentrations of these compounds in otters from Sweden [24].

The present finding of the proposed Müllerian duct remnants could indicate that these otters were exposed to elevated concentrations of estrogens or estrogenic like compounds during fetal development. Unfortunately, there is no documentation from the time period before the increase of EDCs in biota, so it is not possible find out if the prevalence of cysts is increasing or decreasing. However, it would be interesting to study the prevalence of cysts in otters from thriving populations in lower contaminated areas. Also, we cannot rule out possible inbreeding effects after the recovery of the otter population from a historical bottle-neck event in the 1980s. No general geographic pattern can be seen, as otters with cysts are found all over Sweden, as well as otters with no cysts (Figure 2). 

As the otter population is steadily increasing, it is likely that these cysts do not have a negative effect on otter reproduction. Also, rarely other morphologic concomitant abnormalities have been noted in the reproductive tract, making a conclusion of cyst formation etiology difficult. Nevertheless, it would be of interest to further examine the possibility that the mothers of otters with cysts were exposed to estrogen or estrogen-like compounds during pregnancy, as well as alternative explanations to the high prevalence of cysts, such as hereditary factors.

## Supporting Information

Table S1 **Data on otters included in the study.** Identification number, year of death, cause of death, total length (from nose to tip of the tail), weight, age group and locality data are given, as well as number of cysts on each individual. The otters were divided into three age groups according to their estimated age: juvenile (up to 5 months old), subadult (approximately 5 -18 months old) and adult (over 18 months old), based on the presence/absence of long bone growth plates, and in some cases also on the size of the testis and baculum. Some otters lack information on length and/or weight, most often due to severe damages in traffic.
(DOCX)
