# Supplementary material for: High Prevalence of Proposed Müllerian Duct Remnant Cysts on the Spermatic Duct in Wild Eurasian Otters (Lutra lutra) from Sweden
Source: PLoS One. 2013 Dec 23;8(12):e84660. doi: 10.1371/journal.pone.0084660 (PMC3871573; doi:10.1371/journal.pone.0084660)
Supplement: Table S1 — Data on otters included in the study. Identification number, year of death, cause of death, total length (from nose to tip of the tail), weight, age group and locality data are given, as well as number of cysts on each individual. The otters were divided into three age groups according to their estimated age: juvenile (up to 5 months old), subadult (approximately 5 -18 months old) and adult (over 18 months old), based on the presence/absence of long bone growth plates, and in some cases also on the size of the testis and baculum. Some otters lack information on length and/or weight, most often due to severe damages in traffic. (DOCX) [file pone.0084660.s001.docx]

Table S1. Data on otters included in the study. Identification number, year of death, cause of death, total length (from nose to tip of the tail), weight, age group and locality data are given, as well as number of cysts on each individual. The otters were divided into three age groups according to their estimated age: juvenile (up to 5 months old), subadult (approximately 5 -18 months old) and adult (over 18 months old), based on the presence/absence of long bone growth plates, and in some cases also on the size of the testis and baculum. Some otters lack information on length and/or weight, most often due to severe damages in traffic.

| Nr | Year | Cause of death | Total length (cm) | Weight (kg) | Age group | Close village/town | latitude | longitude | No of cysts |
| --- | --- | --- | --- | --- | --- | --- | --- | --- | --- |
| A1999/5116 | 1999 | Traffic | 100 | 6,215 | Subadult | Edsbyn | 61 20.539 | 15 34.605 | 3 or more |
| A2001/8338 | 2001 | Traffic |  | 6,055 | Adult | Åmots-Bruk, Ockelbo | 60 57.816 | 16 26.9320 | 3 or more |
| C2001/5223 | 2001 | Traffic | 107 | 6,68 | Adult | Nunisvaara | 67 8.010 | 20 44.251 | 0 |
| C2002/5377 | 2002 | Traffic | 108 | 7,767 | Adult | Seskarö, Kalix, Haparanda | 65 43.366 | 23 44.155 | 0 |
| A2004/5019 | 2003 | Traffic | 73 | 2,249 | Juvenile | Kvarnåsen, Lycksele, Norsjö | 64 56.515 | 19 4.651 | 0 |
| A2006/5151 | 2003 | Starved | 110 | 5,363 | Adult | Lyckeberg, by lake Glysjön, Eksjö | 57 39.064 | 14 56.167 | 2 |
| A2009/5051 | 2003 | Traffic | 99,1 | 5,90 | Adult | Svappavara | 67 29.352 | 21 7.290 | 1 |
| A2004/5298 | 2004 | Traffic | 107 | 6,687 | Adult | Springliden, Malö | 65 14.654 | 18 57.923 | 1 |
| A2004/5255 | 2004 | Traffic | 104 | 7,682 | Adult | Mårbäck | 61 12.130 | 14 4.303 | 1 |
| A2004/5256 | 2004 | Traffic | 103 | 7,18 | Adult | Between Oxberg and Mora | 61 4.852 | 14 18.777 | 0 |
| A2005/5006 | 2004 | Traffic | 108 | 8,063 | Adult | Älgered, Bergsjö, Hudiksvall | 62 1.159 | 16 55.779 | 1 |
| A2005/5036 | 2004 | Traffic |  | 6,75 | Adult | Hudiksvall | 61 42.350 | 17 4.392 | 3 or more |
| C2004/5586 | 2004 | Starved | 104,5 | 4,117 | Adult | Nattavaara | 66 47.012 | 20 55.118 | 3 or more |
| A2005/5046 | 2005 | Traffic | 104,1 | 7,37 | Subadult | Kyrkbyn, Tierp | 60 17.730 | 17 27.882 | 3 or more |
| A2005/5007 | 2005 | Traffic | 106 | 7,118 | Subadult | Överkalix | 66 24.826 | 22 38.749 | 3 or more |
| A2005/5037 | 2005 | Traffic | 98,5 | 4,844 | Subadult | Bollnäs, Lottefors | 61 26.125 | 16 24.103 | 3 or more |
| A2005/5045 | 2005 | Traffic | 102,1 | 5,91 | Adult | Övsjön, Kälarna | 63 1.638 | 15 56.514 | 2 |
| A2005/5049 | 2005 | Starved | 107,5 | 3,9 | Adult | Handöl | 63 15.472 | 12 27.435 | 3 or more |
| A2005/5051 | 2005 | Traffic | 105 | 6,322 | Adult | Ljusdal | 61 53.008 | 15 56.265 | 2 |
| A2005/5157 | 2005 | Traffic | 100,5 | 6,994 | Adult | Svenstavik | 62 45.144 | 14 26.047 | 2 |
| A2005/5211 | 2005 | Traffic | 109,5 | 7,06 | Adult | Ovansjö kronopark, Kungsberg-Stockbro | 60 43.825 | 16 24.365 | 0 |
| A2005/5215 | 2005 | Traffic | 104 | 7,212 | Adult | Långbanken, between Sollerön and Gesunda | 60 53.609 | 14 33.129 | 3 or more |
| A2005/5217 | 2005 | Traffic |  | 8,388 | Adult | Växjö, Danneberg | 56 54.156 | 14 47.712 | 3 or more |
| A2005/5219 | 2005 | Traffic |  | 7,598 | Adult | Sandviken, Högbo | 60 41.568 | 16 49.011 | 3 or more |
| A2005/5223 | 2005 | Traffic | 98 | 6,084 | Subadult | Kramfors | 62 54.169 | 17 49.925 | 0 |
| A2005/5292 | 2005 | Traffic | 110,7 | 8,2 | Adult | Blommeröd | 55 52.585 | 13 30.355 | 0 |
| A2005/5296 | 2005 | Traffic | 104,9 | 8 | Adult | Lydinge | 59 59.054 | 17 56.732 | 1 |
| A2007/5002 | 2005 | Traffic | 103,1 | 6,4 | Subadult | Bölerakan, Sveden, Bollnäs | 61 17.064 | 16 30.971 | 2 |
| A2007/5246 | 2005 | Traffic | 104 | 4,558 | Adult | Tännäs | 62 26.171 | 12 42.856 | 0 |
| A2006/5158 | 2006 | Traffic | 118,5 | 7,115 | Adult | Engelbrektsgatan, Örebro | 59 16.347 | 15 12.991 | 3 or more |
| A2006/5218 | 2006 | Traffic | 111 | 7,699 | adult | Gumma Kvarn, Eksjö | 57 37.819 | 14 58.122 | 0 |
| A2006/5254 | 2006 | Traffic | 115,9 | 7,4 | adult | Lurbovägen, Norrbo | 60 59.251 | 16 34.589 | 3 or more |
| A2006/5259 | 2006 | Traffic | 105 | 6,05 | adult | Vallnäs, Eksjö | 57 37.250 | 15 28.319 | 2 |
| A2006/5394 | 2006 | Traffic | 106 | 7,944 | Adult | Lima, Malung | 60 56.004 | 13 21.901 | 3 or more |
| A2006/5396 | 2006 | Traffic | 108,5 | 7,806 | Adult | Lilla Vi, Vimmerby | 57 46.132 | 15 51.009 | 0 |
| A2007/5019 | 2006 | Traffic | 118 | 9,737 | Adult | Between Tångån and Hyttöavfarten, Uppsala | 60 30.406 | 17 18.267 | 0 |
| A2007/5020 | 2006 | Traffic | 117 | 8,42 | Adult | Hedåsen, Sandviken Bångsbro | 60 33.357 | 16 45.376 | 3 or more |
| A2007/5025 | 2006 | Traffic | 103,5 | 5,425 | Subadult | Östersund, Häggenäs | 63 23.730 | 15 0.407 | 1 |
| A2007/5026 | 2006 | Traffic | 114 | 7,877 | Adult | Hackås, Riksten | 62 55.069 | 14 31.804 | 1 |
| A2007/5039 | 2006 | Unknown | 109 | 7,214 | Subadult | Boxholm | 58 2.098 | 15 7.469 | 2 |
| A2007/5076 | 2006 | Starved | 119,4 | 5,3 | Adult | Fyrisån, Ulltuna | 59 49.166 | 17 40.115 | 1 |
| A2007/5090 | 2006 | Traffic | 110,2 | 7 | Adult | Bräcke | 62 51.131 | 15 28.936 | 1 |
| A2007/5168 | 2006 | Traffic | 108,9 | 7,7 | Adult | Hindriksheden, Mora | 61 1.411 | 14 36.252 | 2 |
| A2007/5175 | 2006 | Bycaught |  | 11,4 | Adult | Hansjö, Orsa | 61 8.266 | 14 37.514 | 3 or more |
| C2006/5093 | 2006 | Traffic | 97 | 6,402 | Subadult | Jokkmokk | 66 36.809 | 19 51.930 | 3 or more |
| A2007/5027 | 2007 | Traffic | 105 | 8,542 | Adult | Klevhult | 57 20.287 | 14 6.176 | 3 or more |
| A2007/5091 | 2007 | Bycaught | 108,5 | 7,3 | Subadult | Västerdalälven, Dala-Järna, Vansbro | 60 32.592 | 14 21.060 | 1 |
| A2007/5153 | 2007 | Traffic | 114 | 8,53 | Adult | Vissbole, Norrtälje | 59 46.725 | 18 48.991 | 3 or more |
| A2007/5184 | 2007 | Starved | 118,9 | 6,1 | Adult | Gillberga, Edebo, Hallstavik | 60 0.493 | 18 33.888 | 0 |
| A2007/5187 | 2007 | Traffic | 99,5 | 5,8 | Subadult | Gryttjons | 61 47.736 | 16 20.290 | 1 |
| A2007/5222 | 2007 | Traffic | 108 | 6,676 | Adult | Hordal, Byvalla | 60 21.020 | 16 24.476 | 1 |
| A2007/5244 | 2007 | Traffic | 96 | 4,605 | Subadult | Våckelberget, Balungstrand, Falun | 60 54.581 | 15 43.298 | 0 |
| A2007/5247 | 2007 | Traffic | 109 | 6,993 | Adult | Åby, Lagan, Ljungby | 56 54.637 | 14 0.694 | 3 or more |
| A2007/5253 | 2007 | Traffic | 101 | 7,307 | Adult | Skärsta | 61 45.222 | 16 9.651 | 2 |
| A2007/5294 | 2007 | Traffic |  | 8,7 | Adult | Nyborg, Jokkmokk | 66 36.169 | 19 51.482 | 3 or more |
| A2007/5316 | 2007 | Traffic | 115,4 | 8,4 | Adult | Bladåker, Knutby, Sundet | 59 59.074 | 18 14.007 | 0 |
| A2007/5389 | 2007 | Traffic |  |  | Subadult | Ekolsund-Bålsta Övergran, Håbo | 59 34.265 | 17 32.968 | 0 |
| A2007/5433 | 2007 | Traffic | 111 | 7,517 | Subadult | Edsjult Säteri | 57 34.231 | 15 12.090 | 0 |
| A2007/5434 | 2007 | Traffic | 109 | 8,607 | Adult | Bruzaholm, Eksjö | 57 38.467 | 15 17.146 | 2 |
| A2008/5002 | 2007 | Traffic | 116,5 | 9,624 | Adult | Solbacka, Norrtälje | 59 46.270 | 18 44.206 | 3 or more |
| A2008/5029 | 2007 | Traffic | 100,2 | 5,9 | Adult | Hansehultsjön, Vimmerby | 57 38.400 | 15 45.883 | 0 |
| C2009/8585 | 2008 | Traffic | 99 | 4,00 | Adult | Vinberg, Ågård, Falkenberg | 56 57.094 | 12 32.16 | 2 |
| A2008/5430 | 2008 | Traffic | 108 | 8,33 | Adult | Stenselet, Storuman | 65 3.903 | 17 5.683 | 3 or more |
| A2008/5129 | 2008 | Traffic | 102,9 | 7,78 | Adult | Korsnäsvägen, Falun | 60 36.087 | 15 39.410 | 1 |
| A2008/5182 | 2008 | Traffic | 101 | 6,587 | Adult | Himmelsätta, Mockfjärd, Borlänge | 60 32.821 | 15 4.330 | 3 or more |
| A2008/5190 | 2008 | Traffic | 109 | 7,73 | Subadult | Eksjö | 57 38.710 | 14 54.059 | 2 |
| A2008/5217 | 2008 | Traffic |  | 5,1 | Subadult | Hyttön | 60 30.162 | 17 18.462 | 0 |
| A2008/5221 | 2008 | Bycaught | 104 | 6,357 | Subadult | Stora Länsan, Borlänge | 60 29.716 | 15 16.074 | 0 |
| A2008/5222 | 2008 | Bycaught | 96,5 | 5,279 | Subadult | Stora Länsan, Borlänge | 60 29.694 | 15 16.068 | 0 |
| A2008/5278 | 2008 | Unknown |  | 6,79 | Subadult | Haparanda harbour, Nikkala | 65 50.00 | 24 8.682 | 2 |
| A2008/5291 | 2008 | Traffic |  |  | Subadult | Möbyholm, Lista, Eskilstuna | 59 19.051 | 16 17.801 | 1 |
| A2008/5324 | 2008 | Traffic | 108 | 8,243 | Adult | Östersund | 62 59.390 | 14 35.733 | 0 |
| A2008/5331 | 2008 | Traffic | 104,5 | 6,856 | Adult | Höviken, Falun | 60 46.429 | 15 52.730 | 3 or more |
| A2008/5337 | 2008 | Traffic | 102 | 6,201 | Adult | Björna, Nyliden, Örnsköldsvik | 63 42.811 | 18 29.697 | 2 |
| A2008/5338 | 2008 | Traffic | 110,5 | 7,077 | Adult | Bjästa, Örnsköldsvik | 63 12.605 | 18 30.764 | 3 or more |
| A2008/5370 | 2008 | Traffic | 114,4 | 9,2 | Adult | Surehammar | 59 42.550 | 16 12.478 | 3 or more |
| A2008/5372 | 2008 | Traffic | 95,3 | 3,8 | Subadult | Sollerön, Lerön | 60 53.840 | 14 34.011 | 3 or more |
| A2008/5373 | 2008 | Traffic | 107,4 | 4,4 | Adult | Malung | 60 33.607 | 13 32.545 | 3 or more |
| A2008/5405 | 2008 | Traffic | 108 | 7,207 | Adult | Rosvik | 65 26.186 | 21 41.018 | 2 |
| A2008/5408 | 2008 | Traffic | 120 | 9,922 | Adult | Vendel | 60 10.127 | 17 33.707 | 2 |
| A2008/5412 | 2008 | Traffic |  |  | Subadult | Åkeborondellen, Vimmerby | 57 38.894 | 15 48.050 | 3 or more |
| A2008/5413 | 2008 | Traffic |  | 6,8 | Adult | Vetlanda | 57 23.761 | 14 51.365 | 1 |
| A2008/5419 | 2008 | Traffic | 101 | 6,732 | Subadult | Stöde, Sundsvall | 62 25.468 | 16 35.199 | 0 |
| A2008/5426 | 2008 | Traffic | 111 | 9,01 | Adult | Björna, Örnsköldsvik | 63 28.880 | 18 34.721 | 1 |
| A2009/5001 | 2008 | Trauma | 119,5 | 9,40 | Adult | Rosersberg, Upplands Väsby | 59 34.349 | 17 54.819 | 0 |
| A2009/5008 | 2008 | Traffic | 108,5 | 6,57 | Subadult | Abborrträsk, Arvidsjaur | 65 26.782 | 19 22.472 | 1 |
| A2009/5015 | 2008 | Traffic | 102 | 5,98 | Adult | Bollnäs | 61 16.822 | 16 31.293 | 3 or more |
| A2009/5016 | 2008 | Traffic | 99 | 6,19 | Subadult | Kilafors | 61 12.496 | 16 44.634 | 2 |
| A2009/5028 | 2008 | Traffic | 120 | 10,10 | Adult | Vallentuna | 59 33.022 | 18 5.819 | 1 |
| A2009/5313 | 2008 | Traffic | 91,5 | 6,50 | Subadult | Edefors, Harads | 66 13.879 | 20 52.239 | 1 |
| A2010/5031 | 2009 | Traffic | 104 | 7,425 | Adult | Ekhultet, Eksjö | 57 42.919 | 15 0.405 | 3 or more |
| A2009/5287 | 2009 | Traffic | 115 | 4,28 | Adult | Högland, Dorotera | 64 34.233 | 15 51.588 | 3 or more |
| A2009/5307 | 2009 | Traffic | 110 | 6,41 | Adult | Edebro, Hallstavik | 60 3.857 | 18 31.438 | 3 or more |
| A2010/5001 | 2009 | Traffic | 106 | 6,21 | Adult | Örnsköldsvik | 63 16.616 | 18 42.553 | 2 |
| A2010/05091 | 2009 | Traffic | 97,9 | 6,5 | Adult | Högsby, Stora Klo | 57 6.745 | 15 48.792 | 1 |
| A2009/5422 | 2009 | Traffic | 113,6 | 8,90 | Adult | Forsmark | 60 22.130 | 18 9.329 | 3 or more |
| A2010/05296 | 2009 | Traffic | 58,5 | 1,21 | Juvenile | Dala-Husby, Hanåker | 60 23.034 | 16 4.064 | 0 |
| A2009/5005 | 2009 | Traffic | sva | 9,50 | Adult | Vadbron, Bergshamra, Norrtälje | 59 38.080 | 18 37.266 | 2 |
| A2009/5011 | 2009 | Bycaught | 91 | 4,34 | Subadult | Rimforsa | 58 8.538 | 15 41.675 | 0 |
| A2009/5020 | 2009 | Traffic | 110,5 | 8,90 | Adult | Estuna church | 59 49.115 | 18 39.094 | 3 or more |
| A2009/5054 | 2009 | Killed | 106,5 | 4,50 | Adult | Backsjö, Junsele | 63 43.063 | 16 45.028 | 1 |
| A2009/5127 | 2009 | Traffic | 105,5 | 6,89 | Adult | Between Sjövik and Sävsjö | 57 26.210 | 14 42.344 | 3 or more |
| A2009/5140 | 2009 | Bycaught | 82 | 2,72 | Juvenile | Between Hermanboda and Ånge | 62 32.522 | 15 49.377 | 0 |
| A2009/5150 | 2009 | Traffic |  | 9,50 | Adult | Älandsbro, Härnösand | 62 42.379 | 17 52.080 | 0 |
| A2009/5308 | 2009 | Bycaught | 106 | 6,97 | Subadult | Ringsjön, Höör | 55 52.930 | 13 32.832 | 0 |
| A2009/5316 | 2009 | Bycaught | 104,7 | 6,40 | Subadult | Lake Liljan, Bengtsheden, Falun | 60 42.319 | 15 52.911 | 0 |
| A2009/5340 | 2009 | Traffic | 108 | 6,27 | Adult | Hedemora | 60 15.609 | 16 0.876 | 1 |
| A2009/5359 | 2009 | Traffic | 111 |  | Subadult | Arboga | 59 24.383 | 15 45.539 | 0 |
| A2009/5361 | 2009 | Traffic |  | 7,80 | Adult | Linköping | 58 24.391 | 15 37.913 | 1 |
| A2009/5388 | 2009 | Traffic | 100 | 6,40 | Subadult | Fagersta | 59 56.243 | 15 49.278 | 0 |
| A2009/5389 | 2009 | Traffic | 115,8 | 8,90 | Subadult | Between Bromölla and Gualöv | 56 3.258 | 14 26.600 | 2 |
| A2009/5391 | 2009 | Traffic | sva | 6,70 | Adult | Värnäs, Torsby | 60 25.892 | 13 15.447 | 1 |
| A2009/5401 | 2009 | Traffic | 97 | 5,65 | Subadult | Gråska, Hallstavik | 60 5.435 | 18 28.936 | 0 |
| A2009/5402 | 2009 | Traffic | 118 | 9,04 | Subadult | Hållsta, Eskilstuna | 59 16.590 | 16 24.880 | 0 |
| A2009/5403 | 2009 | Traffic |  | 8,732 | Adult | Ingtorp-Mariannelynd | 57 38.085 | 15 25.263 | 0 |
| A2009/5408 | 2009 | Unknown |  |  | Subadult | Östersund | 63 9.663 | 14 39.170 | 0 |
| A2009/5409 | 2009 | Starved | 97,5 | 3,86 | Subadult | Revsund, Bräcke, Östersund | 62 54.041 | 15 7.508 | 0 |
| A2009/5413 | 2009 | Traffic | 99 | 6,19 | Adult | Arnäsväll, Örnsköldsvik | 63 18.370 | 18 54.624 | 1 |
| A2009/5415 | 2009 | Traffic |  | 6,86 | Subadult | Linköping | 58 26.083 | 15 31.805 | 1 |
| A2009/5417 | 2009 | Traffic | 104,2 | 5,30 | Adult | Delsbo | 61 47.902 | 16 33.072 | 3 or more |
| A2009/5442 | 2009 | Starved |  | 4,80 | Adult | Krokum, Landögssjön | 63 35.810 | 14 3.059 | 0 |
| A2009/5444 | 2009 | Traffic |  | 7,60 | Adult | Tranås | 57 59.302 | 14 40.910 | 1 |
| A2009/5449 | 2009 | Traffic | 115,5 | 8,99 | Subadult | Onsäng, Söderhamn | 61 14.908 | 17 2.009 | 3 or more |
| A2009/5451 | 2009 | Traffic | 97,5 | 5,10 | Subadult | Sollerön, Orsa | 60 54.018 | 14 35.050 | 3 or more |
| A2009/5463 | 2009 | Traffic |  | 7,00 | Adult | Gesala, Västerås | 59 43.042 | 16 34.838 | 2 |
| A2009/5468 | 2009 | Starved |  | 2,224 | Juvenile | Överkalix | 66 19.998 | 22 49.164 | 1 |
| A2010/05090 | 2009 | Traffic | 102,3 | 6,7 | Adult | Jämljög | 61 45.425 | 16 27.881 | 1 |
| A2010/05191 | 2009 | Traffic | 107,2 | 6,7 | Adult | Delsbro, Gräddviksvallen | 61 45.425 | 16 27.881 | 2 |
| A2010/5072 | 2009 | Traffic | 97,5 | 7,233 | Adult | Arvidsjaur | 65 35.395 | 19 31.795 | 2 |
| A2010/05270 | 2010 | Traffic | 104 | 7,094 | Subadult | Tollarp, Kristianstad | 55 55.772 | 13 58.576 | 1 |
| A2010/05265 | 2010 | Unknown |  | 8,40 | Adult | Munkån, Lindberg | 57 10.024 | 12 18.188 | 3 or more |
| A2010/05428 | 2010 | Traffic | 102,5 | 5,8 | Adult | Linghed, Falun | 60 46.750 | 15 52.482 | 3 or more |
| A2010/05297 | 2010 | Traffic | 97 | 6,879 | Subadult | Luleå, Älvsbyn | 65 39.221 | 21 13.818 | 1 |
| A2010/05468 | 2010 | Traffic | 112 | 9,332 | Adult | Gullringen, Vimmerby | 57 50.737 | 15 44.823 | 3 or more |
| A2010/05396 | 2010 | Traffic |  | 9,725 | Adult | Täby, Karlbo | 59 29.004 | 18 3.046 | 0 |
| A2010/05322 | 2010 | Unknown |  |  | Subadult | Northen Sweden, no exact locality |  |  | 0 |
| A2010/05469 | 2010 | Traffic | 94 | 4,058 | Subadult | Källan, Transtrand, Sälen | 61 4.923 | 13 19.565 | 0 |
| A2010/05215 | 2010 | Traffic |  | 6,4 | Subadult | Vassunda, Knivsta, Brantshammarsån | 59 43.666 | 17 43.221 | 3 or more |
| A2010/05308 | 2010 | Traffic | 102 | 5,396 | Adult | Matfors | 62 20.896 | 17 1.618 | 2 |
| A2010/05354 | 2010 | Bycaught | 96,8 | 4,48 | Subadult | Lake Orsa | 61 6.505 | 14 37.189 | 1 |
| A2010/05386 | 2010 | Traffic | 96 | 6,304 | Subadult | Västbygge, Venjan | 60 56.940 | 13 53.392 | 2 |
| A2010/05394 | 2010 | Traffic | 112 | 7,153 | Subadult | Stavabacken, Åkersberga | 59 29.594 | 18 14.663 | 1 |
| A2010/05475 | 2010 | Bycaught | 126 | 10,205 | Adult | Södra Vi, Vimmerby | 57 45.734 | 15 54.938 | 2 |
| A2010/05507 | 2010 | Traffic | 112,3 | 9,2 | Adult | Ulvsberg, Gamlekil, Linköping, Nykil | 58 15.816 | 15 23.151 | 2 |
| A2010/05515 | 2010 | Traffic | 114 | 8,165 | Adult | Skebobruk, Västertorpet | 59 58.363 | 18 36.133 | 0 |
| A2010/05534 | 2010 | Traffic | 105,1 | 5,89 | Subadult | Neistenkangas, Rantakangas | 66 52.413 | 23 55.792 | 2 |
| A2010/05536 | 2010 | Traffic |  | 6,5 | Subadult | Söderfors, Tierp | 60 23.291 | 17 14.844 | 0 |
| A2010/05546 | 2010 | Bycaught | 115,5 | 8,407 | Adult | Långrammen, Västervik | 57 50.484 | 16 15.288 | 2 |
| A2011/05594 | 2010 | Traffic | 89 | 3,7 | Subadult | Torsböle, between Örnsköldsvik and Gideå | 63 22.583 | 18 53.131 | 0 |
| A2010/05221 | 2010 | Traffic | 101 | 6,286 | Subadult | Haningeleden, Linköping | 58 23.171 | 15 37.750 | 3 or more |
| A2010/05321 | 2010 | Traffic | 83,5 | 2,992 | Subadult | Revässaari, Nikkala, Haparanda | 65 47.211 | 23 50.093 | 0 |
| A2010/05426 | 2010 | Traffic | 108,6 | 7,2 | Adult | Råneå | 65 52.803 | 22 22.850 | 2 |
| A2010/05431 | 2010 | Traffic | 110,4 |  | Adult | Between Olofström and Åkeholm, Svängsta | 56 17.210 | 14 42.580 | 0 |
| A2010/05432 | 2010 | Traffic | 111,4 | 7,85 | Adult | Karlsbyheden, Falun | 60 38.886 | 15 50.606 | 1 |
| A2010/05433 | 2010 | Traffic | 118,4 | 9,3 | Adult | Brobacken, Nykrogen | 60 2.770 | 16 20.508 | 2 |
| A2010/05449 | 2010 | Traffic | 116,5 | 9,816 | Adult | Ekeby, Kumla | 59.1588 | 15.0777 | 3 or more |
| A2010/05465 | 2010 | Traffic |  | 8,2 | Subadult | Vallmotorp, Katriheholm, Sundtorp | 59 1.711 | 16 12.842 | 3 or more |
| A2010/05500 | 2010 | Traffic | 105,6 | 6,9 | Adult | Nykrogen | 60 2.771 | 16 20.510 | 1 |
| A2010/05514 | 2010 | Traffic |  |  | Adult | Humlebäcken | 56 8.446 | 12 55.789 | 0 |
| A2010/05537 | 2010 | Traffic |  | 7,1 | Adult | Söderfors, Tierp | 60 23.291 | 17 14.844 | 1 |
| A2011/05283 | 2010 | Traffic | 107,2 | 6,4 | Subadult | Kopparnäs | 65 23.953 | 21 28.062 | 0 |
| A2011/05331 | 2010 | Bycaught |  | 5,9 | Adult | Finnaryd, by lake Söljen, Jönköping | 57 55.464 | 14 53.386 | 1 |
| A2011/05334 | 2010 | Traffic |  | 7,2 | Subadult | Näsum, Bromölla | 56 9.701 | 14 25.601 | 1 |
| C2011/00001 | 2010 | Killed | 115,8 | 6,1 | Adult | Tibro, by the river Tidan | 58 25.334 | 14 8.9 | 3 or more |
| A2011/05464 | 2011 | Traffic | 101,6 | 6,8 | Adult | Lappträsk, Sangis | 65 58.943 | 23 27.826 | 2 |
| A2011/05246 | 2011 | Traffic | 109 | 8,618 | Adult | Sund, Södra Vi, Vimmerby | 57 44.854 | 15 49.855 | 1 |
| A2011/05425 | 2011 | Traffic | 97 | 5,347 | Adult | Skräddarbo, Ovanåker | 59 29.276 | 18 14.832 | 2 |
| A2011/05426 | 2011 | Traffic | 104,5 | 4,859 | Subadult | Bollnäs | 61 11.211 | 15 52.582 | 0 |
| A2011/05443 | 2011 | Trauma | 103,5 | 6,848 | Subadult | Galtsjön, Ronneby | 56 13.244 | 15 12.601 | 1 |
| A2012/05077 | 2011 | Traffic | 117 | 9,672 | Adult | Odengatan, Jönköping | 57 46.658 | 14 11.727 | 0 |
| A2012/05080 | 2011 | Traffic | 99,5 | 5,518 | Subadult | Bastusel, Arvidsjaur | 65 37.356 | 19 44.815 | 0 |
| A2011/05251 | 2011 | Bycaught | 122 | 10,965 | Adult | Tämmaren, Helganbo, Östervåla | 60 9.985 | 17 13.820 | 0 |
| A2011/05029 | 2011 | Traffic | 110,3 | 7,009 | Subadult | Eskilstuna | 59 8.816 | 16 42.763 | 0 |
| A2011/05237 | 2011 | Traffic | 111 | 8,11 | Adult | Mörsil | 63 18.663 | 13 39.112 | 2 |
| A2011/05247 | 2011 | Traffic | 107 | 9,278 | Subadult | Åsljunga, Örkeljunga | 56 18.25 | 13 23.59 | 0 |
| A2011/05258 | 2011 | Traffic |  | 8,6 | Adult | Söderön, Öregrund, Tvärnö | 60 13.2 | 18 29.313 | 1 |
| A2011/05285 | 2011 | Traffic | 136 | 9,1 | Adult | Valbo, Gävle | 60 38.872 | 17 1.648 | 3 or more |
| A2011/05432 | 2011 | Traffic | 121 | 9,56 | Adult | Råby-Rönö, Edstorp | 58 52.504 | 16 52.762 | 0 |
| A2011/05459 | 2011 | Bycaught |  | 7,4 | Subadult | Vuollerim, River Stora Luleälven | 66 29.264 | 20 34.849 | 0 |
| A2011/05461 | 2011 | Traffic | 106,5 | 7,751 | Adult | Årsundavägen, Sandviken, Härsnäs | 60 34.272 | 16 45.321 | 3 or more |
| A2011/05473 | 2011 | Traffic | 108,5 | 8,1 | Adult | N Lake Sommen, Tranås | 58 8.966 | 14 58.936 | 3 or more |
| A2011/05533 | 2011 | Traffic | 105,5 | 6,508 | Subadult | Hästveda, Hässleholm | 56 16.496 | 13 57.737 | 1 |
| A2011/05569 | 2011 | Traffic | 108,5 | 8,362 | Subadult | Korsberga, Vetlanda | 57 18.353 | 15 7.487 | 2 |
| A2011/05596 | 2011 | Traffic | 114 | 7,819 | Adult | Kusböle, Myrviken | 53 0.2041 | 14 8.0511 | 3 or more |
| A2012/05041 | 2011 | Traffic | 112,2 | 9,5 | Adult | Hallersrum-Bullerbo, Djursdala, Vimmerby | 57 46.824 | 15 51.373 | 1 |
| A2011/05102 | 2011 | Traffic | 106,5 | 8,345 | Subadult | Värnamo, Bor | 57 6.391 | 14 10.191 | 1 |
| A2011/05252 | 2011 | Traffic |  |  | Subadult | South of Lake Sommen, Bäck | 57 58.624 | 15 3.028 | 0 |
| A2011/05253 eller A2012/05253 | 2011 | Bycaught |  |  | Adult | Aneby | 57 49.435 | 14 48.799 | 1 |
| A2011/05257 | 2011 | Traffic |  | 8,9 | Adult | Gimo | 60 10.548 | 18 11.527 | 2 |
| A2011/05282 | 2011 | Traffic | 111,6 |  | Adult | Råbo, Hudiksvall | 61 49.102 | 17 1.935 | 2 |
| A2011/05289 | 2011 | Traffic |  |  | Adult | Eksjö | 57 37.818 | 14 58.135 | 0 |
| A2011/05333 | 2011 | Traffic |  | 6,1 | Adult | Vännäsby | 63 55.271 | 19 51.283 | 0 |
| A2011/05438 | 2011 | Bycaught | 108 | 7,716 | Adult | Malviken, Häggdånger, Härnösand | 62 29.778 | 17 48.130 | 3 or more |
| A2011/05463 | 2011 | Bycaught | 116 | 10,4 | Adult | Hamrångeån, Norrsundet | 60 55.379 | 17 1.924 | 3 or more |
| A2011/05467 | 2011 | Traffic | 100,2 | 5,4 | Subadult | Djupdal, Hudiksvall | 61 45.471 | 16 47.361 | 0 |
| A2011/05469 | 2011 | Traffic | 103,5 | 6 | Adult | Gävle kommun | 60 38.664 | 17 1.253 | 0 |
| A2011/05472 | 2011 | Traffic | 104,5 | 6,5 | Adult | Rundviksvägen | 63 32.933 | 19 24.559 | 2 |
| A2011/05474 | 2011 | Traffic | 111 | 7,3 | Adult | Stångåkajen, Linköping | 58 24.943 | 15 37.749 | 1 |
| A2011/05540 | 2011 | Bycaught | 107,3 | 6,8 | Subadult | Lake Mångeln, Ockelbo | 60 50.645 | 16 37.963 | 1 |
| A2011/05557 | 2011 | Traffic | 112 | 9,2 | Adult | Ystad | 55 26.065 | 13 54.277 | 0 |
| A2011/05597 | 2011 | Trauma | 97,6 | 4 | Adult | Mjölksvattsfjället, Kolåsen | 63 52.305 | 13 16.799 | 3 or more |
| A2012/05037 | 2011 | Traffic | 105 | 7 | Adult | Trångfors, Hallstahammar | 59 37.876 | 16 11.880 | 1 |
| A2012/05040 | 2011 | Traffic | 102,6 | 6,4 | Adult | not known |  |  | 3 or more |
| A2012/05042 | 2011 | Traffic | 111,9 | 8 | Adult | Ryssby, Ljungby | 56 51.889 | 14 10.120 | 0 |
| A2012/05829 | 2012 | Starved | 62 | 1,31 | Juvenile | Norrttälje | 59 45.433 | 18 42.344 | 0 |
| A2012/05653 | 2012 | Traffic | 106,5 | 7,935 | Adult | Uppsala | 59 29.785 | 18 9.903 | 1 |
| A2012/05063 | 2012 | Traffic | 106,5 | 6,557 | Subadult | Hoting | 64 7.169 | 16 5.841 | 1 |
| A2012/05082 | 2012 | Traffic | 80,5 | 3,243 | Subadult | Aneby | 57 50.966 | 14 49.511 | 0 |
| A2012/05470 | 2012 | Traffic | 113,6 | 6,9 | Adult | Vilhelmina | 64 56.512 | 15 50.140 | 3 or more |
| A2012/05539 | 2012 | Traffic | 107 | 7,847 | Adult | Åtvidaberg | 58 6.331 | 16 6.710 | 1 |
| A2012/05563 | 2012 | Traffic | 97,5 | 4,4 | Subadult | Eksjö | 57 41.447 | 14 57.335 | 0 |
| A2012/05663 | 2012 | Traffic | 111,5 | 7,2 | Adult | Eksjö area, no exact locality | 57 38.710 | 14 54.059 | 1 |
| A2012/05722 | 2012 | Bycaught | 97,9 | 6,424 | Subadult | Örebro | 59 11.830 | 15 36.487 | 0 |
| A2012/05819 | 2012 | Traffic | 98 | 5,819 | Subadult | Eksjö | 57 38.918 | 149518,00 | 1 |
| A2012/05820 | 2012 | Traffic | 106 | 8,119 | Adult | Hok | 57 30.036 | 14 17.298 | 3 or more |
| A2012/05822 | 2012 | Traffic | 110,5 | 8,51 | Adult | Mjölby, Spångsholm | 58 21.332 | 15 13.103 | 1 |
| A2012/05830 | 2012 | Traffic | 97 |  | Adult | Lake Bölesjön, Njurunda | 62 13.951 | 17 22.462 | 1 |
| A2012/05832 | 2012 | Traffic | 109 | 8,643 | Adult | Umeå | 63 49.578 | 20 19.905 | 2 |
| A2012/5564 | 2012 | Bycaught | 104 | 6,019 | Subadult | Väddöviken, Tomta, Norrtälje | 60 4.658 | 18 45.907 | 2 |
| A2012/05309 | 2012 | Traffic | 88 | 3,9 | Subadult | Falun | 60 36.258 | 15 37.884 | 1 |
| A2012/05361 | 2012 | Traffic | 110,5 | 8,127 | Adult | Alsterån, Ålem | 56 57.286 | 16 23.740 | 2 |
| A2012/05469 | 2012 | Traffic | 110 | 8,7 | Adult | Ryd, Tingsryd | 56 27.877 | 14 42.189 | 2 |
| A2012/05471 | 2012 | Traffic |  |  | Adult | Gimån, Bräcke | 62 48.852 | 15 21.225 | 1 |
| A2012/05666 | 2012 | Bycaught | 97,7 | 5,8 | Subadult | Södra Rängen, Labbenäsviken, Sturefors | 58 18.470 | 15 40.518 | 2 |
| A2012/05671 | 2012 | Traffic | 112,2 | 9,1 | Adult | Klockarbo, Österåker, Vingåker | 59 6.074 | 15 56.809 | 2 |
| A2012/05673 | 2012 | Traffic | 106,5 | 7,4 | Adult | Granån, Robertsfors | 64 13.969 | 20 58.233 | 2 |
| A2012/05688 | 2012 | Traffic |  | 5,7 | Adult | Uppsala | 60 11.648 | 17 52.714 | 1 |
| A2012/05704 | 2012 | Traffic | 105,3 | 5,4 | Adult | Järnforsen, Målilla | 57 24.506 | 15 34.264 | 1 |
| A2012/05715 | 2012 | Traffic | 108,8 | 7,7 | Adult | Figgesund | 61 53.647 | 16 32.536 | 3 or more |
| A2012/05875 | 2012 | Bycaught | 104,2 | 5,2 | Adult | Gavle-Långsjön, Rånäs | 18 18.264 | 18.3044 | 0 |
| A2012/05881 | 2012 | Traffic | 113 | 9 | Adult | Nottebäck, Växjö | 57 5.368 | 15 11.297 | 0 |
| A2013/05005 | 2012 | Traffic |  |  | Subadult | Sikströmmen, Hysingsvik, Norrtälje | 59 40.888 | 18 49.426 | 1 |
| A2013/05016 | 2012 | Traffic |  | 7,6 | Adult | Brokind, Linköping | 58 7.578 | 15 40.476 | 1 |
| A2013/05074 | 2012 | Traffic |  |  | Adult | Vibäcken, Svenstavik | 62 52.234 | 14 31.329 | 1 |
| A2012/05038 | 2011 | Traffic | 107 |  | Adult | Sveg area, no exact locality | 62 1.894 | 14 22.141 | 2 |
